# Causes of stillbirth and death among children younger than 5 years in eastern Hararghe, Ethiopia: a population-based post-mortem study

**DOI:** 10.1016/S2214-109X(23)00211-5

**Published:** 2023-06-01

**Authors:** Lola Madrid, Addisu Alemu, Anna C Seale, Joe Oundo, Tseyon Tesfaye, Dadi Marami, Hiwot Yigzaw, Alexander Ibrahim, Ketema Degefa, Tadesse Dufera, Zelalem Teklemariam, Tadesse Gure, Haleluya Leulseged, Stefanie Wittmann, Mahlet Abayneh, Surafel Fentaw, Fikremelekot Temesgen, Melisachew M Yeshi, Mehret Dubale, Zerihun Girma, Caroline Ackley, Berhanu Damisse, Markus Breines, Stian M S Orlien, Dianna M Blau, Robert F Breiman, Ebba Abate, Yadeta Dessie, Nega Assefa, J Anthony G Scott, CHAMPS Ethiopia Study Group, Merga Deresa, Gezahegn Mengesha, Tigistu Samuel, Eyoel Taye, Mohammed Aliyi, Gurmu Feyissa, Yenenesh Tilahun, Getahun Wakwaya, Simegn Tadesse, Kidish Asnake, Mariamcher Ayalew, Azeb Kidane, Emmanuel Azore, Mussie Berhanu, Mulu Berihun, Mersan Deresa, Nardos Assegid, Shirine Voller, Mahlet Mekonnen, Andualem Alemaheyu, Nana Sarkodie-Mensah, Beth Morrison, Boniface Jibendi, Asnake Worku, Alemenesh Mirkuzie, Fentabil Getnet

**Affiliations:** a Department of Infectious Disease Epidemiology, London School of Hygiene & Tropical Medicine, London, UK; b College of Health and Medical Sciences, Haramaya University, Harar, Ethiopia; c KEMRI-Wellcome Trust Research Programme, Kilifi, Kenya; d Warwick Medical School, University of Warwick, Coventry, UK; e St Paul's Hospital Millennium Medical College, Addis Ababa, Ethiopia; f Ethiopian Public Health Institute, Addis Ababa, Ethiopia; g Addis Ababa University, Addis Ababa, Ethiopia; h Mekele University, Mekele, Ethiopia; i Department of Global Health and Infection, Brighton and Sussex Medical School, Brighton, UK; j Center for Global Health, Centers for Disease Control and Prevention, Atlanta, GA, USA; k Department of Global Health, Rollins School of Public Health, Emory University, Atlanta, GA, USA

## Abstract

**Background:**

Child mortality is high in Ethiopia, but reliable data on the causes of death are scarce. We aimed to gather data for the contributory causes of stillbirth and child deaths in eastern Ethiopia.

**Methods:**

In this population-based post-mortem study, we established a death-notification system in health facilities and in the community in Kersa (rural), Haramaya (rural) and Harar (urban) in eastern Ethiopia, at a new site of the Child Health and Mortality Prevention Surveillance (CHAMPS) network. We collected ante-mortem data, did verbal autopsies, and collected post-mortem samples via minimally invasive tissue sampling from stillbirths (weighing at least 1000 g or with an estimated gestational age of at least 28 weeks) and children who died younger than 5 years. Children—or their mothers, in the case of stillbirths and deaths in children younger than 6 months—had to have lived in the catchment area for the past 6 months to be included. Molecular, microbiological, and histopathological analyses were done in collected samples. Cause of death was established by an expert panel on the basis of these data and classified as underlying, comorbid, or immediate separately for stillbirths, neonatal deaths (deaths aged 0–27 days), and child deaths (aged 28 days to <5 years).

**Findings:**

Between Feb 4, 2019, and Feb 3, 2021, 312 deaths were eligible for inclusion, and the families gave consent in 195 (63%) cases. Cause of death was established in 193 (99%) cases. Among 114 stillbirths, the underlying cause of death was perinatal asphyxia or hypoxia in 60 (53%) and birth defects in 24 (21%). Among 59 neonatal deaths, the most common underlying cause was perinatal asphyxia or hypoxia (17 [29%]) and the most common immediate cause of death was neonatal sepsis, which occurred in 27 (60%). Among 20 deaths in children aged 28 days to 59 months, malnutrition was the leading underlying cause (15 [75%]) and infections were common immediate and comorbid causes. Pathogens were identified in 19 (95%) child deaths, most commonly *Klebsiella pneumoniae* and *Streptococcus pneumoniae*.

**Interpretation:**

Perinatal asphyxia or hypoxia, infections, and birth defects accounted for most stillbirths and child deaths. Most deaths could have been prevented with feasible interventions, such as improved maternity services, folate supplementation, and improved vaccine uptake.

**Funding:**

Bill & Melinda Gates Foundation.

## Introduction

In Ethiopia, the frequency of childhood mortality has declined substantially in the past 15 years in parallel with the introduction of life-saving interventions such as pneumococcal and rotavirus vaccination, but it still remains high.^1^ Estimated mortality in children younger than 5 years (hereafter referred to as under-5 mortality) fell from 123 deaths per 1000 livebirths in 2005 to 55 deaths per 1000 livebirths in 2019.^1^ However, Ethiopia has a population of over 120 million people,^2^ and in 2020 there were still an estimated 172 889 deaths in children younger than 5 years and 90 323 stillbirths.^3^

WHO recommends the use of verbal autopsy to identify causes of death in all age groups in places where no other system is in place for this end.^4^, ^5^ This process involves structured interviews with people close to the deceased. Although much effort has gone into optimising the process, the efficacy of verbal autopsies remains limited by recall bias and by difficulties with distinguishing between diseases with similar clinical presentations. Verbal autopsy data suggest that 62% of under-5 mortality in Ethiopia is due to acute respiratory infections, diarrhoea, preterm birth complications, birth asphyxia, and neonatal sepsis.^6^ In eastern Ethiopia specifically, verbal autopsies have identified neonatal sepsis, birth asphyxia, respiratory disorders, and prematurity as the major causes of death among neonates.^7^

Complete diagnostic autopsy is the most accurate method to ascertain cause of death^8^, but acceptability and capacity issues substantially limit use in resource-constrained settings.^9^ Minimally invasive tissue sampling (MITS), which involves taking percutaneous core tissue biopsies from several organs in the first hours after death, was developed to provide an acceptable alternative to complete diagnostic autopsy.^10^ Data obtained via MITS can be combined with other data, including ante-mortem clinical notes and verbal autopsy, to increase the accuracy of the reported cause of death. Use of MITS has been validated in perinatal and paediatric deaths, and has good agreement with complete diagnostic autopsy per the categories of the tenth revision of the International Classification of Diseases (ICD-10).^10^, ^11^, ^12^

**Research in context**

**Evidence before this study**

We searched PubMed with the terms “cause of death” AND (“Stillbirth” OR “children” OR “neonates”) AND “Ethiopia” for articles published in any language between Jan 1, 2000, and Jan 1, 2021, that included original data on causes of death among stillbirths and children younger than 5 years in Ethiopia. Our search identified 141 articles. By reviewing the abstracts of these articles, we filtered our findings down to nine papers that contained original data for causes of death in children younger than 5 years. Three papers included population-based data (one each from northern, southwestern, and eastern Ethiopia) gathered via verbal autopsy. A validated, WHO-recommended method to ascertain cause of death in low-income settings (involving use of a standard verbal-autopsy questionnaire and either physician coding or a computer algorithm to assign cause of death) was used in only one study (of causes of death among neonates in eastern Hararghe, done by the Kersa Health and Demographic Surveillance System). However, even these methods can be constrained by recall bias, particularly when used to record stillbirths or the deaths of young infants, and thus only broad categories of causes of death can be defined. More detailed information can be obtained via complete diagnostic autopsy, which provides accurate, pathogen-specific diagnoses. However, such autopsies are often not feasible because of resource constraints, lack of local expertise, and lack of acceptability to families and communities in the countries with the highest mortality. The Child Health and Mortality Prevention Surveillance (CHAMPS) network establishes cause of death on the basis of minimally invasive tissue sampling (ie, taking tiny tissue specimens post mortem and doing microbiological and histopathological examinations). This approach improves the accuracy of cause-of-death assessments. Published data from the first five CHAMPS sites in South Africa, Mozambique, Kenya, Bangladesh, and Mali suggest that the main causes of stillbirths were perinatal asphyxia or hypoxia, whereas preterm birth complications caused most neonatal deaths, and birth defects, lower respiratory tract infections, and HIV caused most deaths in children younger than 5 years.

**Added value of this study**

In this CHAMPS study, we used innovative post-mortem minimally invasive tissue sampling—combined with mortality surveillance, clinical information, and verbal autopsy data—to generate for the first time high-quality data for the causes of stillbirth and child death in eastern Hararghe, Ethiopia. We identified preventable causes of death in all age groups, including neural tube defects, hypoxia or perinatal asphyxia, neonatal sepsis, vaccine-preventable disease, and malnutrition. In view of the absence of previous population-based data for causes of death in Ethiopia, our findings provide a rich dataset to guide public health policy.

**Implications of all the available evidence**

Our data are being used to direct public health interventions locally, nationally, and internationally, with the goal of reducing stillbirth and child death. Our results support the introduction of interventions or strategies to improve antenatal care and maternal services, reduce neural tube defects through folate fortification, increase vaccine delivery and uptake, improve infection prevention and control programmes, and improve management of malnutrition in health facilities.

The Child Health and Mortality Prevention Surveillance (CHAMPS) programme collects population-based data for causes of stillbirths and child deaths at a network of sites with high child mortality. The CHAMPS protocol includes use of MITS and ancillary mortality data to provide a detailed analysis of the chain of events leading up to stillbirth or child death rather than identifying a single underlying cause.^13^, ^14^ CHAMPS aims to use its data to inform public health interventions locally, nationally, and internationally and to achieve the Sustainable Development Goal of eliminating preventable childhood deaths. CHAMPS has published results from five established research units with existing morbidity data (in South Africa, Mozambique, Bangladesh, Kenya, and Mali).^15^ Here we present mortality surveillance data from a new CHAMPS site in eastern Ethiopia, a setting where child mortality is high but evidence to understand the causes of death is scarce.^16^

## Methods

### Study site and population

In this population-based study, we collected standardised, population-based, longitudinal data for under-5 mortality and stillbirths between Feb 4, 2019, and Feb 3, 2021, to establish causes of death in eastern Ethiopia within the CHAMPS network. This network includes 12 sites in seven countries in sub-Saharan Africa and south Asia, the regions where most child deaths occur.^17^ Ethiopia was selected as an important country to include in the network because it has a large population and high child mortality.^18^ The CHAMPS site in eastern Ethiopia was considered a green-field site because it had little previous experience of aetiology research^14^ and has been described in detail previously.^16^ In February, 2019, CHAMPS activities started in two health and demographic surveillance system (HDSS) areas in eastern Hararghe: Harar (urban) and Kersa (rural). A third area, Haramaya (rural) was added to CHAMPS activities in June, 2020. These three HDSS areas collectively formed the catchment area for this study

Harar is the capital of the Harari region and is located 520 km east of Addis Ababa. The Harar HDSS was established in 2012, and includes six health centres that provide delivery services and primary health care. In 2019, the resident population was 58 095, and the under-5 mortality was between five and 20 deaths per 1000 livebirths.^16^ Kersa is 44 km from Harar. The Kersa HDSS was established in September, 2007.^19^ In 2019, the resident population of the Kersa HDSS was 136 505 and there were 86 deaths among under-5s per 1000 livebirths.^16^ The Kersa HDSS catchment area includes six health centres that provide primary health care, including deliveries. Haramaya is located between Kersa and Harar. The HDSS was set up in 2019, and started surveillance for pregnancies and deaths in January, 2020. Its resident population is 93 313, and the under-5 mortality is anticipated to be similar to that in Kersa HDSS. The Haramaya HDSS catchment area has six health centres providing primary health care (including deliveries). At Haramaya district hospital, which serves the populations of Haramaya and Kersa, there are more than 3000 deliveries and around 1700 paediatric admissions each year.

Hiwot Fana Comprehensive Specialized Hospital (HFCSH), the regional referral hospital, is located in Harar and serves 5·8 million people, including the populations of Haramaya, Harar, and Kersa HDSSs. At HFCSH, there are approximately 3000 paediatric admissions annually, half of which are neonates, and around 5500 deliveries per year, 9% of which result in stillbirth. Roughly a fifth of the neonatal admissions are preterm babies. Leading causes of paediatric admission at HFCSH are pneumonia, measles, meningitis, and malnutrition. Among neonates, the most frequent reasons for admission are sepsis and birth asphyxia (Health Management Information System data; unpublished).

In HDSS areas, births, deaths, migration, marital status, and pregnancy are monitored through biannual household visits. In this study, we focused on stillbirths (which we defined as no spontaneous breathing or movement at the time of delivery of infants weighing at least 1000 g or with an estimated gestational age of at least 28 weeks) and deaths in children younger than 5 years in the Haramaya, Harar, and Kersa HDSSs. We considered neonates (ie, children who died aged 0–27 days) and older children (ie, those who died aged 28 days to 59 months) separately. Children—or their mothers, in the case of stillbirths and deaths in children younger than 6 months—had to have lived in the catchment area for the past 6 months to be included in the study. Deaths were considered eligible for investigation by MITS if the body was available for post-mortem sampling and the death had occurred within the past 24 h (or the past 36 h if the body had been refrigerated).

This study was approved and renewed annually by the Haramaya University ethics committee, the National Ethics Committee in Ethiopia (MoSHE 04/246/661/21), and the London School of Hygiene & Tropical Medicine research ethics committee (ref 14394). Written informed consent was provided by the deceased's caregiver before all study post-mortem investigations.

### Mortality surveillance and data collection

The CHAMPS sites conduct mortality surveillance independently of the intermittent HDSS mortality surveillance. The mortality surveillance began on Feb 4, 2019, and targeted stillbirths and child deaths among residents of the Harar and Kersa HDSSs. In Kersa, we implemented a death-notification system via mobile phones. Deaths occurring at health centres were reported by health-care workers and those occurring in the community by trained notifiers (religious or community leaders, health extension workers, influential women, such as traditional healers, and HDSS data collectors). After notification, study counsellors, supported by the trained notifiers and the clinical research team, approached the family of the deceased and provided counselling. If the family provided consent, the deceased was transported in a study vehicle, accompanied by the family, to the autopsy room in Kersa Health Centre, where all MITS was done in that area.

In Harar, a hospital-based death notification system was established at HFCSH through a call centre that linked hospital staff who identified and reported deaths with research staff. The research staff assessed study eligibility and checked for duplications. If the deaths were eligible for inclusion, they approached the family of the deceased and provided counselling. If the family provided consent, the MITS procedure was done in the autopsy room at the hospital. If the death occurred outside the hospital, a study vehicle was used to transport the body and the family from the place of death to the hospital. Death notification in Haramaya HDSS began on June 12, 2020. When Haramaya HDSS was developed, we replicated the hospital-based death notification system from HFCSH in Haramaya Hospital and introduced the community-based death notification system developed in Kersa HDSS to Haramaya HDSS. The MITS procedure was done in the autopsy room in Haramaya Hospital.

### Procedures

Data for each death, including dates of birth and death, contact information, and a description of the circumstances of the death, were collected on standardised forms and entered into a REDcap database. For deaths identified in health facilities, data were abstracted from clinical records. Verbal autopsies were done within 1 month of the death. Regular quality reports were generated and reviewed by the local data manager and the CHAMPS network statistician to ensure the quality of all study data.

MITS was done by trained staff. The procedure consisted of photographs, assessment of dysmorphic features, anthropometric measurements, post-mortem minimally invasive tissue biopsies, and specimen collection. Non-tissue specimens included blood and cerebrospinal fluid (CSF), stool samples (collected by rectal brush), and a nasopharyngeal swab specimen. Biopsy needles were used to collect tissue specimens from the lungs, brain, liver, and placenta (if available), as described elsewhere.^20^ All samples were sent to the local laboratory (at the College of Health and Medical Sciences, Harar) for processing.

The laboratory in Harar cultured post-mortem blood samples and CSF, checked for HIV RNA by PCR, and did malaria thick and thin smears and rapid diagnostic tests for malaria and sickle cell anaemia. TaqMan Array Cards (ThermoFisher Scientific, Waltham, MA, USA) with specific molecular assays were used to detect multiple pathogens. Respiratory samples (ie, lung and nasopharyngeal specimens) were tested for 39 pathogens, stool samples for 32 pathogens, and blood and CSF samples for 49 pathogens.^21^ All tissues were examined using standard histopathological techniques, including routine stains and special stains, such as tissue Gram stain. Immunohistochemistry was done by the Central Pathology Laboratory at the US Centers for Disease Control and Prevention (CDC; Atlanta, GA, USA), when relevant, as described elsewhere.^20^ Aliquots of formalin-fixed tissue specimens were shipped three times a year from the local laboratory to the CDC. Histopathology was done jointly by pathologists at the HFCSH histopathology laboratory and the CDC.

The cause of death for a case was reviewed by a determination of cause of death (DeCoDe) panel when MITS was done and all relevant data were collected. The panel consisted of local microbiologists, obstetricians, pathologists, paediatricians, and public health experts who were trained to interpret the chain of events leading up to death by using two reference guidelines: the ICD-10 and the WHO application of ICD-10 to deaths during the perinatal period.^22^, ^23^ Panel members reviewed all available information for each case, including clinical records, ante-mortem and maternal diagnostic data if available, post-mortem photographs, anthropometric measurements, post-mortem microbiology and histopathology results, and the findings of verbal autopsies, and confirmed that the eligibility criteria for post-mortem sampling were met. They then assigned an underlying cause to all deaths, which was defined as the condition that initiated the chain of events that led to the death. Deaths for which no cause was identified were classified as undetermined. When more than one condition was considered to contribute to death, the entire chain of events was documented, including underlying, comorbid conditions (in the causal pathway between underlying and immediate causes) and immediate causes (including maternal factors when relevant). Under the DeCoDe procedure, only one underlying cause of death and one immediate cause can be established, but there can be multiple comorbid causes.

Specific causes of death were grouped into broader categories for analysis (appendix pp 3–5). The panel classified the deaths as preventable or not preventable on the basis of the availability of effective public health interventions and provided recommendations to families, health facilities, and health authorities for each case. We used medians, counts, and percentages to summarise data, and calculated 95% CIs to provide estimates of uncertainty (given the small sample size of the population of interest). All analyses were done in Stata/BE (version 17.0) and R (version 3.6.3).

### Role of the funding source

The funder of the study contributed to study design but had no role in data collection, data analysis, data interpretation, or writing of the report.

## Results

Between Feb 4, 2019, and Feb 3, 2021, 1369 deaths were registered in the three HDSS areas, including 173 stillbirths and 1196 deaths among children younger than 5 years (appendix p 6). 773 (56%) of these deaths occurred outside health facilities. Overall, there were 15 stillbirths per 1000 births (10 per 1000 in both Haramaya HDSS and Harar HDSS, and 19 per 1000 in Kersa HDSS) and 109 under-5 deaths per 1000 livebirths (44 per 1000 in Haramaya, 21 per 1000 in Harar, and 157 per 1000 in Kersa; appendix p 6).

During the same period, the hospital-based and community-based death-notification systems detected 2237 stillbirths or deaths among children younger than 5 years. 536 (24%) of these deaths occurred among residents of the study catchment area (figure). 183 of these deaths occurred at home (appendix p 7). The hospital-based and community-based death-notification systems therefore identified 39% (ie, 536 of 1369) of the deaths that occurred in the catchment area.

**Figure:**
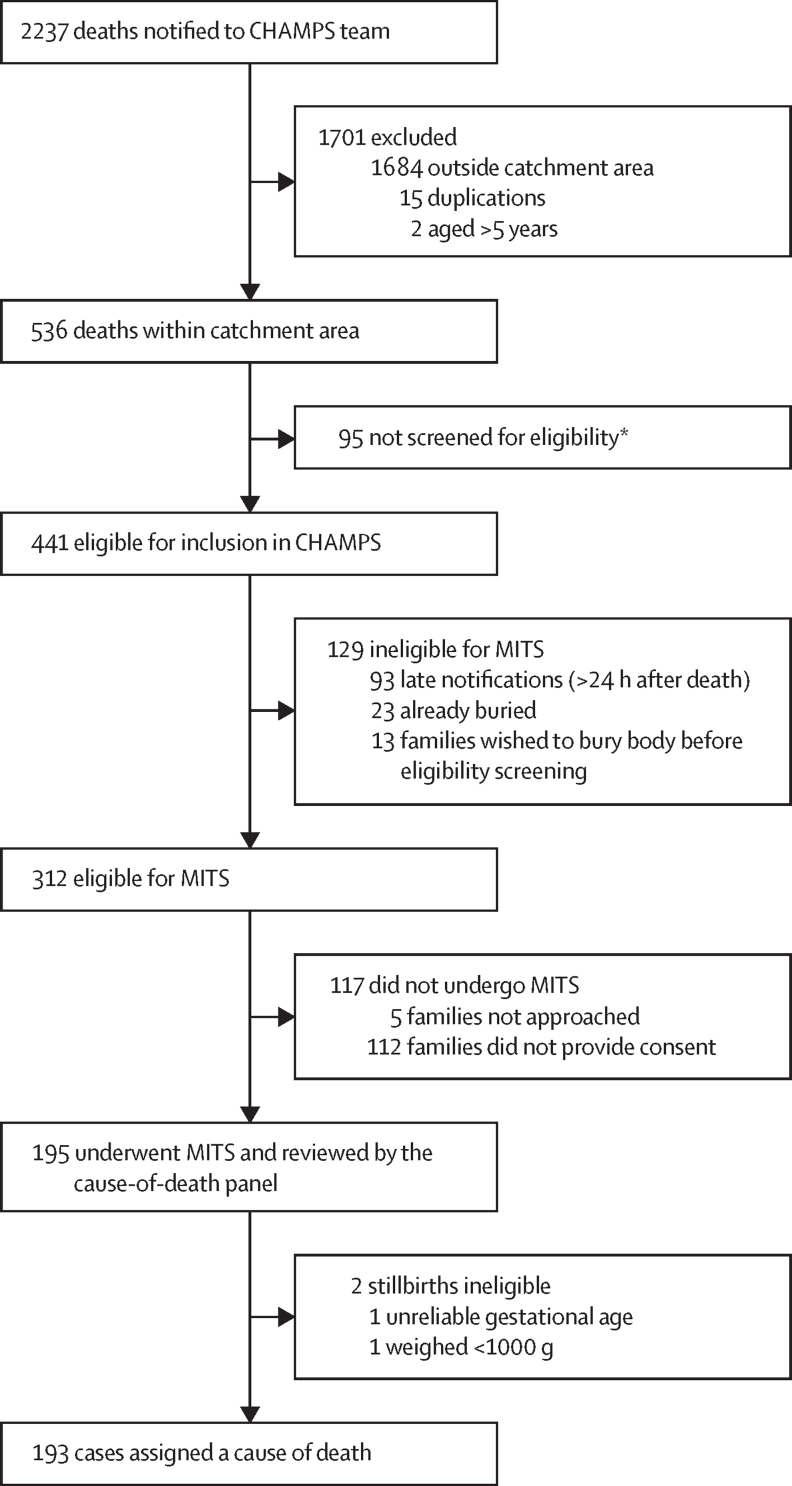
Overview of inclusion in CHAMPS analysis of cause of death CHAMPS=Child Health and Mortality Prevention Surveillance. MITS=minimally invasive tissue sampling. *Reasons for not screening for eligibility included political instability, being unable to reach the family, and complex family dynamics.

441 (83%) of the 536 death notifications were eligible for inclusion. However, 129 (29%) were unsuitable for MITS, mostly because of delay in the notification of death (figure). 119 (92%) of the deaths excluded were recorded in Kersa, compared with six (5%) in Haramaya and four (3%) in Harar. 96 (74%) were home deaths. 25 (19%) were stillbirths and 36 (28%) were neonatal deaths.

Among the remaining 312 deaths who were eligible for MITS, only 46 (15%) died at home. The families of 195 (63%) of the deceased gave consent, and MITS was done. In general, consent for post-mortem investigation was obtained more frequently for stillbirths than for deaths in children and was obtained more readily for deaths identified in health facilities than those identified in the community (appendix p 8).

Two stillbirths in whom MITS was done were retrospectively excluded because they weighed less than 1000 g (n=1) or had a gestational age of less than 28 weeks (n=1). Thus, 193 eligible deaths were reviewed by the DeCoDe panel—14% of the 1369 deaths captured by the HDSS during the same period. 52 (27%) of the deaths reviewed occurred in the Haramaya HDSS area, 83 (43%) in the Harar HDSS area, and 58 (30%) in the Kersa HDSS area. Median time from death to MITS was 3·25 h (IQR 1·40–10·15). 173 (90%) of the 193 deaths occurred at health facilities (table 1). At least one cause of death was identified in 180 (93%) cases, and two or more comorbid conditions were identified in 80 (42%). In 57 (30%) deaths, at least one contributory pathogen was identified.

**Table 1:** Sociodemographic and clinical characteristics of deaths reviewed by cause-of-death panel, by health and demographic surveillance system site

	**Haramaya (n=52)**	**Harar (n=83)**	**Kersa (n=58)**
**Age at death**			
Stillbirth	40 (77%)	44 (53%)	30 (52%)
<24 h	5 (10%)	19 (23%)	4 (7%)
1–6 days	5 (10%)	15 (18%)	5 (9%)
7–27 days	1 (2%)	2 (2%)	3 (5%)
28 days to <12 months	1 (2%)	3 (4%)	5 (9%)
12 months to <5 years	0	0	11 (19%)
**Sex**			
Male	24 (46%)	47 (57%)	34 (59%)
Female	28 (54%)	36 (43%)	24 (41%)
**Ethnicity**			
Oromo	32 (62%)	39 (47%)	45 (78%)
Amhara	0	12 (14%)	2 (3%)
Other	0	5 (6%)	0
Unknown or no response	20 (38%)	27 (33%)	11 (19%)
**Religion**			
Muslim	52 (100%)	40 (48%)	55 (95%)
Ethiopian Orthodox	0	31 (37%)	3 (5%)
Other	0	9 (11%)	0
Unknown	0	3 (4%)	0
**Location of death**			
Facility	51 (98%)	82 (99%)	40 (69%)
Community	1 (2%)	1 (1%)	18 (31%)
**Time to MITS**			
≤24 h	52 (100%)	82 (99%)	58 (100%)
<36 h	0	1 (1%)	0

Data are n (%). MITS=minimally invasive tissue sampling.

A cause of death was identified in 101 (89%) of 114 stillbirths (table 2). The placenta was available for examination in 47 (41%) cases. Perinatal asphyxia or hypoxia and birth defects were the most frequently identified underlying cause of death (table 2). Among 24 stillbirths associated with birth defects, neural tube defects were reported in 19: eight (42%) had craniorachischisis, five (26%) had spina bifida, three (16%) had anencephaly, and three (16%) had iniencephaly. A pathogen was judged to have contributed to stillbirth in three (3%) cases, two in which *Streptococcus agalactiae* was the only isolate and one case of polymicrobial infection with *Escherichia coli* and *S agalactiae* (table 3). A maternal factor was a contributing factor in 59 (52%) stillbirths; the most common factors were complications of the placenta, cord, or membranes (27 [46%]), maternal hypertensive disorders or diabetes (19 [32%]), and complications during labour and delivery (eight [14%]; appendix p 9).

**Table 2:** Causes of death among stillbirths

		**Underlying cause of death (n=114)**
Non-infectious congenital conditions		
	Perinatal asphyxia or hypoxia	60 (53% [43–62])
	Small for gestational age	11 (10% [4–15])
	Other*	3 (3% [0–6])
Birth defects		
	Neural tube defects	19 (17% [10–24])
	Other†	5 (4% [1–8])
Sepsis		3 (3% [0–6])
Undetermined		13 (11% [6–17])

Data are n (% [95% CI]). Data for the immediate cause of death were available in 17 cases (all caused by perinatal asphyxia or hypoxia) and for comorbid cause of death in two cases (congenital hydrocephalus [n=2]).

* Hydrops fetalis (n=2) and low birthweight (n=1).

† Congenital hydrocephalus (n=1), Down syndrome (n=1), congenital malformations syndrome predominantly affecting facial appearance (n=1), congenital malformation of the musculoskeletal system (n=1), and osteochondrodysplasia with defect of growth of tubular bones and spine (n=1).

**Table 3:** Pathogens identified in causal chain leading to death, by condition and age group

	**Total number of isolates**	**Underlying cause of death**	**Immediate cause of death**	**Comorbid cause of death**	**Sepsis**	LRTI*	**Meningitis**	**Gastrointestinal diseases**	**Other**	Cases in age group*
**Stillbirths (n=114)**										
*Streptococcus agalactiae*	3	3	0	0	3	0	0	0	0	3 (3%)
*Escherichia coli*	1	1	0	0	1	0	0	0	0	1 (1%)
**Neonates (n=59)**										
*Klebsiella pneumoniae*	59	5	26	28	29	16	14	0	0	30 (51%)
*Salmonella* species	11	1	4	6	5	1	5	0	0	6 (10%)
*E coli*	9	1	6	2	7	0	0	2	0	7 (12%)
*Haemophilus influenzae*	6	2	2	2	2	3	1	0	0	3 (5%)
*S agalactiae*	3	0	1	2	1	1	1	0	0	1 (2%)
*E coli* or *Shigella* species†	2	0	2	0	2	0	0	0	0	1 (2%)
*Enterobacter cloacae*	2	0	2	0	2	0	0	0	0	2 (3%)
*Enterococcus* species	2	0	1	1	1	1	0	0	0	2 (3%)
*Candida albicans*	2	0	1	1	1	0	1	0	0	1 (2%)
*Moraxella catarrhalis*	2	2	0	0	0	2	0	0	0	2 (3%)
*Toxoplasma gondii*	1	1	0	0	0	0	0	0	1	1 (2%)
*Ureaplasma* species‡	1	0	1	0	1	0	0	0	0	1 (2%)
*Streptococcus pneumoniae*	1	1	0	0	0	1	0	0	0	1 (2%)
*Acinetobacter baumanii*	1	0	0	1	0	1	0	0	0	1 (2%)
*Staphylococcus aureus*	1	1	0	0	0	1	0	0	0	1 (2%)
*Klebsiella terragina*	1	0	1	0	1	0	0	0	0	1 (2%)
*Pseudomonas aeruginosa*	1	0	0	1	0	1	0	0	0	1 (2%)
*Streptococcus* species‡	1	0	1	0	1	0	0	0	0	1 (2%)
**Infants and children (n=20)**										
*K pneumoniae*	18	0	7	11	8	8	2	0	0	11 (55%)
*S pneumoniae*	16	0	8	8	5	9	2	0	0	9 (45%)
*H influenzae*	10	0	4	6	4	4	2	0	0	7 (35%)
*E coli*	11	1	4	6	6	0	2	3	0	6 (30%)
*Streptococcus pyogenes*	6	0	1	5	2	1	3	0	0	2 (10%)
*P aeruginosa*	5	0	2	3	2	2	1	0	0	2 (10%)
*Neisseria meningitidis*	3	1	2	0	1	0	2	0	0	2 (10%)
Measles virus	2	1	0	1	0	0	0	0	2	2 (10%)
*A baumanii*	2	0	0	2	0	2	0	0	0	2 (10%)
Cytomegalovirus	2	0	1	1	1	1	0	0	0	1 (5%)
Respiratory syncytial virus	1	1	0	0	0	1	0	0	0	1 (5%)
*Salmonella* species	2	0	1	1	0	0	1	1	0	1 (5%)
*Vibrio cholerae*	1	0	1	0	0	0	0	1	0	1 (5%)
*C albicans*	1	0	1	0	1	0	0	0	0	1 (5%)
*Pneumocystis jirovecii*	1	0	0	1	0	1	0	0	0	1 (5%)
Influenza virus type B	1	0	1	0	0	1	0	0	0	1 (5%)
*Enterococcus* species‡	1	0	0	1	0	0	0	1	0	1 (5%)

Data are n or n (%). LRTI=lower respiratory tract infection.

* Data are for the proportion of children with each infection by age group.

† TaqMan Array Cards (ThermoFisher Scientific, Waltham, MA, USA) are unable to distinguish *E coli* from *Shigella* species, and thus in instances when other microbial tests were negative, it was not possible to definitively identify the genus.

‡ TaqMan Array Cards cannot distinguish specific species of these genera, and thus when other microbial tests were negative, we could not specify the species.

A specific cause of death was identified for all 59 neonates reviewed by the panel (table 4). The most common underlying causes were perinatal asphyxia or hypoxia, preterm birth, and related complications such as respiratory distress (table 4). One immediate or one or more comorbid additional causes were assigned to 45 (76%) neonatal deaths (median 2 [IQR 1–4]). The most common immediate cause was neonatal sepsis, and the most common comorbid conditions were lower respiratory tract infections and meningitis (table 3). At least one pathogen was identified as a contributor to death in 35 (59%) neonates, and more than one pathogen was implicated in 14 (24%) deaths. *Klebsiella pneumoniae* was identified in 30 (51%) neonatal deaths, *E coli* in seven (12%) and *Salmonella* species in six (10%; table 3).

**Table 4:** Causes of death among neonates

	**Underlying cause of death (n=59)**	**Immediate cause of death (n=45)**	Comorbid cause of death (n=72)*
**Non-infectious congenital conditions**			
Perinatal asphyxia or hypoxia	17 (29% [17–40])	3 (7% [0–14])	4 (6% [0–11])
Low birthweight	5 (8% [1–16])	0	7 (10% [3–17])
Hypothermia	0	0	12 (17% [8–25])
Meconium aspiration syndrome	4 (7% [0–13])	6 (13% [3–23])	1 (1% [0–4])
Other†	1 (2% [0–5])	3 (7% [0–14])	2 (3% [0–7])
Hypoglycaemia	0	0	3 (4% [0–9])
**Infectious diseases**			
Sepsis	4 (7% [0–13])	27 (60% [46–74])	2 (3% [0–7])
Lower respiratory tract infections	3 (5% [0–11])	2 (4% [0–10])	17 (24% [14–33])
Meningitis	0	1 (2% [0–7])	16 (22% [13–32])
Congenital toxoplasmosis	1 (2% [0–5])	0	0
**Preterm birth complications**			
Respiratory distress syndrome	7 (12% [4–20])	2 (4% [0–10])	2 (3% [0–7])
Prematurity	9 (15% [6–24])	0	0
Necrotising enterocolitis	0	0	2 (3% [0–7])
Intraventricular haemorrhage	0	0	2 (3% [0–7])
Other‡	1 (2% [0–5])	0	1 (1% [0–4])
**Birth defects**			
Neural tube defects	2 (3% [0–8])	0	0
Other§	5 (8% [1–16])	1 (2% [0–7])	1 (1% [0–4])

Data are n (% [95% CI]).

* Includes 32 neonates with at least one comorbid cause of death and 22 neonates with more than one comorbid cause of death.

† Underlying cause: accidental suffocation (n=1); immediate cause: kernicterus (n=1), hypoxic ischaemic encephalopathy (n=1), and neonatal jaundice (n=1); comorbid cause: kernicterus (n=1) and disseminated intravascular coagulation (n=1).

‡ Underlying cause: apnoea of prematurity (n=1); comorbid cause: jaundice of prematurity (n=1).

§ Underlying cause: congenital malformation syndrome predominantly affecting facial appearance (n=2), polycystic kidney (n=1), absence of unspecified limbs (n=1), and unspecified chromosomal abnormality (n=1); immediate cause: congenital hydrocephalus (n=1); comorbid cause: congenital hydrocephalus (n=1).

Of the 20 children younger than 5 years who died after the neonatal period, malnutrition was the underlying cause of death in 15 (75%), including 12 (60%) with severe acute malnutrition (ie, with marasmus or kwashiorkor, or marasmic kwashiorkor; table 5). In 19 (95%), one immediate or one or more comorbid causes of death was assigned (median 4 [IQR 3–5]), most commonly sepsis (table 4). 36 comorbid conditions were identified, of which lower respiratory tract infections and meningitis were the most common occurring (table 5). At least one pathogen was found in 19 (95%) child deaths, and in 13 (65%) cases more than one pathogen was identified. *K pneumoniae* was isolated in 11 (55%) and *Streptococcus pneumoniae* in nine (45%; table 3).

**Table 5:** Causes of death among children aged 1–59 months

	**Underlying cause of death (n=20)**	**Immediate cause of death (n=19)**	Comorbid cause of death (n=36)*
**Infectious diseases**			
Sepsis	1 (5% [0–15])	11 (58% [36–80])	4 (11% [1–21])
Lower respiratory tract infections	1 (5% [0–15])	4 (21% [3–39])	11 (31% [16–46])
Meningitis	0	1 (5% [0–15])	9 (25% [11–39])
Diarrhoeal diseases	1 (5% [0–15])	2 (11% [0–24])	4 (11% [1–21])
Measles	1 (5% [0–15])	0	1 (3% [0–8])
**Malnutrition disorders**			
Marasmus	6 (30% [10–50])	0	0
Kwashiorkor	6 (30% [10–50])	0	0
Other†	3 (15% [0–31])	0	1 (3% [0–8])
**Other child disorders**			
Anaemia	0	0	2 (6% [0–13])
Liver diseases	1 (5% [0–15])	0	1 (3% [0–8])
Hypovolaemic shock	0	1 (5% [0–15])	0
Neurological disorders	0	0	1 (3% [0–8])
Skin and subcutaneous diseases	0	0	1 (3% [0–8])
Disseminated intravascular coagulation	0	0	1 (3% [0–8])

Data are n (% [95% CI]).

* Includes 17 children with at least one comorbid cause of death and 11 with more than one comorbid cause of death.

† Underlying cause: low birthweight (n=2) and marasmic kwashiorkor (n=1); comorbid cause: stunting (n=1).

Most of the conditions in the category “non-infectious congenital and conditions” were identified as unique causes of death. In stillbirths, this category was dominated by perinatal asphyxia or hypoxia and congenital birth defects (appendix pp 10–11). Preterm birth complication was a common underlying condition in neonates, and was linked to meningitis, sepsis, and non-infectious conditions (appendix p 12). Among children who died aged 28 days to 59 months, malnutrition triggered comorbid and immediate causes of death—mostly sepsis, lower respiratory tract infections, and meningitis (appendix p 12).

## Discussion

To our knowledge, ours is the first study in Ethiopia that integrates post-mortem MITS with ante-mortem data to characterise the specific causes of stillbirth and under-5 mortality both at home and in health facilities. Perinatal asphyxia or hypoxia was the most common underlying cause of death among stillbirths and neonates. Beyond the neonatal period, nutritional disorders were the leading underlying cause of death. A contributory pathogen was identified in 95% of children aged 28 days to 59 months and 59% of neonates. *K pneumoniae* was the most frequently isolated pathogen in neonates and children younger than 5 years.

Our findings about stillbirth are similar to those from other CHAMPS sites, where perinatal asphyxia or hypoxia was the most common underlying cause of death.^15^ However, unlike at other CHAMPS sites, at this site in eastern Hararghe, Ethiopia, congenital birth defects (mostly neural tube defects), were the second most common cause of stillbirth. The high prevalence of neural tube defects has already been noted in hospital-based surveillance in Ethiopia.^24^, ^25^ Periconceptional folate supplementation is not widely used in Ethiopia and there is no mandatory food-fortification programme, both of which would probably reduce this burden.

Among neonatal deaths, perinatal asphyxia or hypoxia, sepsis, and preterm birth complications were the main causes of death, in line with findings from other CHAMPS sites. A similar pattern was apparent in Kersa HDSS data derived from verbal autopsy alone^7^ and in a previous hospital-based study of causes of death in Ethiopia.^15^, ^26^ Neonatal sepsis was mostly caused by *K pneumoniae* in our study, consistent with findings in other reports from Ethiopia^27^ and elsewhere,^15^ highlighting the need for more tools to prevent *Klebsiella* sepsis.

Among non-neonates younger than 5 years, nutritional disorders were the most common underlying cause of death in our study. This finding contrasts with those from other CHAMPS sites,^15^ where infections including HIV, malaria, and lower respiratory tract infections were more common. At our study site in eastern Hararghe, neither HIV nor malaria were endemic but malnutrition has previously been described as one of the main causes of child deaths.^19^ At the Ethiopian site, as at other CHAMPS sites, immediate and comorbid conditions in the chain of events were mostly infectious diseases.^15^

Our study has four important limitations. First, the sample of deaths investigated differed from the overall sample of deaths recorded in the HDSS on two relevant criteria: we studied fewer deaths that occurred at home and fewer deaths among children aged 28 days to 59 months. Consequently, our findings might not fully reflect the population in which the deaths occurred. The data presented here for these under-represented groups are insufficient to provide a population-weighted overview of the main causes of death, but future additional sampling specifically targeted at under-represented groups should rectify this issue. Second, the role of infection as a contributor to death is difficult to interpret when many pathogens are found simultaneously in a single infant. The DeCoDe panel reviewed all the data available for each death and decided that an infection contributed to death only when evidence (largely based on pathology findings) was sufficient to support such a conclusion. Furthermore, the histopathological and immunohistochemistry procedures used in our study could distinguish infection accompanied by an inflammatory response from post-mortem superinfection, and the fact that the post-mortem examinations were done rapidly after death reduced the risk of post-mortem overgrowth.^28^ Third, we did not do genetic tests, micronutrient tests, or other investigations that could help to identify congenital disorders and inborn errors of metabolism, so these causes are not represented in our results. Finally, the DeCoDe process is susceptible to human errors and group psychology, which can result in inconsistency and a lack of objectivity. To reduce the influence of these factors, the panel followed a coordinated set of standard operating procedures linked to the ICD-10 guidelines,^22^, ^23^ which are used across all CHAMPS sites. Furthermore, 10% of cases from all sites are reviewed by all the panels at other sites as a quality assurance exercise.^29^ In future, algorithmic pathways informed by artificial intelligence could help to standardise these DeCoDe decisions.

Despite these limitations, this novel mortality surveillance system has generated initial baseline data that could focus further research to generate actionable data for policy makers to reduce child mortality locally and nationally in the near future in Ethiopia. We have used our findings to start to work with stakeholders to improve obstetric and antenatal care, encourage research and preventive actions to reduce neural tube defects across Ethiopia through food-fortification programmes, increase vaccination coverage, implement effective infection prevention and control programmes in hospitals to reduce nosocomial infections (particularly those caused by *K pneumoniae*), and improve management of malnutrition, which was an underlying cause of death in cases with varied immediate causes.

## Data sharing

Summarised data are publicly available via CHAMPS. Access to the full data set for research and evaluation purposes can be made directly to CHAMPS.

## Declaration of interests

ACS has been affiliated with the Bill & Melinda Gates Foundation (the study funder) since October, 2023 (after this manuscript was first submitted). This affiliation is not directly related to the submitted work. All other authors declare no competing interests.
