# Supplementary material for: Causes of stillbirth and death among children younger than 5 years in eastern Hararghe, Ethiopia: a population-based post-mortem study
Source: Lancet Glob Health. 2023 Jun 1;11(7):e1032–40. doi: 10.1016/S2214-109X(23)00211-5 (PMC10282072; doi:10.1016/S2214-109X(23)00211-5)
Supplement: Supplementary appendix [file mmc1.pdf]

THE LANCET
Global Health

Supplementary appendix

This appendix formed part of the original submission and has been peer reviewed. We post it as supplied by the authors.

Supplement to: Madrid L, Alemu A, Seale AC, et al. Causes of stillbirth and death among children younger than 5 years in eastern Hararghe, Ethiopia: a population-based post-mortem study. *Lancet Glob Health* 2023; published online June 1. https://doi.org/10.1016/S2214-109X(23)00211-5.

Appendix

Causes of stillbirth and death among young children in Eastern Hararghe, Ethiopia: a population-based post-mortem study.

Table of Contents

Table S1. Causes of death and International Classification of Diseases (ICD-10) codes (grouped into broader categories) 3

Table S2. Maternal factors contributing to main cause of death in stillbirths and International Classification of Diseases (ICD-10) codes (grouped into broader categories) 5

Table S3. Frequency of stillbirths, neonatal and child deaths in the target population (HDSS) that are enrolled in the study and consented for MITS (4th February 2019 to 3rd February 2021). 6

Figure S1: Death notification diagram 7

Table S4. Breakdown of consent by HDSS, age at death and location of death 8

Figure S2A: Categories of all identified causes of death combined for stillbirths, neonates, and children under 5 years 10

Figure S2B: Categories of all identified causes of death and position in the causal chain, combined for stillbirths, neonates, and children under 5 years 11

Figure S3. Causal chain of events of all identified causes of death, combined for stillbirths, neonates, and children under 5 years 12

Table S1. Causes of death and International Classification of Diseases (ICD-10) codes (grouped into broader categories)

Category	Condition	ICD-10
**Undetermined**	Stillbirths	P95
	Neonates and children	R99
**Preterm birth complications**	Preterm	P07.3
	Extreme immaturity	P07.2
	Respiratory distress syndrome	P22.0
	Necrotizing enterocolitis of foetus and newborn	P77
	Intraventricular haemorrhage	P52.2
	Neonatal jaundice associated with preterm delivery	P59.0
**Congenital birth defects**	Chromosomal abnormality, unspecified	Q99.9
	Congenital hydrocephalus	Q03.9
	Congenital malformation-absence of unspecified limbs	Q73.0
	Congenital malformation syndromes predominantly affecting facial appearance	Q87.0
	Neural Tube Defect-Craniorachischisis	Q00.1
	Neural Tube Defect-lumbar spina bifida without hydrocephalus	Q05.7
	Neural Tube Defect-lumbar spina bifida with hydrocephalus	Q05.2
	Neural Tube Defect-thoracic spina bifida with hydrocephalus	Q05.1
	Neural Tube Defect-Anencephaly	Q00.0
	Neural Tube Defect-Iniencephaly	Q00.2
	Polycystic kidney disease	Q61.3
	Osteochondrodysplasia with defect of growth of tubular bones and spine	Q77
	Congenital malformation of the musculoskeletal system	Q79
	Congenital malformations syndrome predominantly affecting facial appearance	Q87
	Downs Syndrome, unspecified	Q90.9
**Congenital and neonatal infections**	Congenital toxoplasmosis	P37.1
	Congenital pneumonia	P23.6
**Non-infectious congenital and neonatal conditions**	Intrauterine hypoxia	P20.1
	Extremely low birth weight	P07.0
	Other low birth weight	P07.1
	Meconium aspiration syndrome	P24.0
	Light for gestational age	P05.0
	Slow foetal growth	P05.9
	Small for gestational age	P05.1
	Hydrops fetalis not due to haemolytic disease	P83.2
	Hydrops fetalis due to other and unspecified haemolytic disease	P56.9
	Birth asphyxia, severe	P21.0
	Birth asphyxia, moderate	P21.1
	Birth asphyxia, unspecified	P21.9
	Hypoxic ischemic encephalopathy of newborn	P91.6
	Other apnoea of the newborn	P28.4

	Disseminated intravascular coagulation	P60
	Kernicterus	P57.0
**Malnutrition**	Marasmus or severe protein-energy malnutrition	E41 - E43
	Kwashiorkor	E40
	Marasmic kwashiorkor	E42
	Moderate protein-energy malnutrition	E44.0
	Stunting	E45
**Sepsis**	Sepsis due to other specified pathogen	A41.8
	Bacterial sepsis of the newborn due to other specific pathogens	P36.8
	Bacterial sepsis of the newborn due to group B Streptococcus	P36.0
	Sepsis due to group A Streptococcus	A40.0
	Sepsis due to other Gram negatives organisms	A41.5
	Sepsis due to Salmonella	A02.1
	Bacterial sepsis of the newborn due to *Escherichia coli*	P36.4
	Sepsis, unspecified	A41.9
**Diarrheal diseases**	Gastroenteritis/Enteritis due to Enteroinvasive *Escherichia coli*	A04.2
	Gastroenteritis/Enteritis due to Enteropathogenic *Escherichia coli* (EPEC)	A04.0
	Gastroenteritis/Enteritis due to Enterotoxigenic *Escherichia coli* (ETEC)	A04.1
	Cholera due to *Vibrio cholerae*	A00.0
	Other unspecified gastroenteritis	A09.0
**Lower respiratory tract infections**	Pneumonia due to Respiratory Syncytial virus	J12.1
	Pneumonia due to *S. pneumoniae*	J13
	Pneumonia due to *Klebsiella spp.*	J15.0
	Pneumonia due to group B Streptococcus	J15.3
	Pneumonia due to other Gram negatives organisms	J15.6
	Pneumonia due to other specified bacteria	J16.8
	Aspiration pneumonia	J69.0
**Meningitis/Encephalitis**	Meningococcal meningitis	A39.0
	Pneumococcal meningitis	G00.1
	Streptococcal meningitis	G00.2
	Meningitis due to other specified bacterial	G00.8
**Other diseases**	Portal hypertension	K76.6
	Acute and subacute hepatic failure	K72.0
	Measles	B05
	Accidental suffocation or strangulation in bed	W75
	Hypovolemic shock	R57.1
	Disseminated intravascular coagulation	D65
	Febrile convulsions	R56.0
	Hypothermia	T68
	Anaemia	D64
	Hypoglycaemia	E16.2
	Hydrocephalus, unspecified	G91.9
	Cellulitis or abscess of the mouth	K12.2

Table S2. Maternal factors contributing to main cause of death in stillbirths and International Classification of Diseases (ICD-10) codes (grouped into broader categories)

Category	Maternal factor	ICD-10
**Foetus affected by complications of placenta, cord, and membranes**	Foetus and newborn affected by other forms of placental separation and haemorrhage (Abruption placentae)	P02.1
	Foetus affected by other and unspecified morphological and functional abnormalities of placenta	P02.2
	Foetus and newborn affected by other specified complications of labour and delivery	P03.8
	Premature rupture of membranes, onset of labour after 24 hours	O42.1
	Premature rupture of membranes, labour delayed by therapy	O42.2
	Foetus and newborn affected by prolapsed cord	P02.4
	Foetus and newborn affected by placental transfusion syndromes	P02.3
	Foetus and newborn affected by chorioamnionitis	P02.7
	Prolonged pregnancy	O48
	Foetus and newborn affected by oligohydramnios	P01.2
**Foetus and newborn affected by multiple pregnancy**	Foetus and newborn affected by multiple pregnancy	P01.5
**Uterine rupture during delivery**	Uterine rupture during delivery	O71.1
**Foetus and newborn affected by other malpresentation, malposition, disproportion and other specified complications during labour and delivery**	Foetus and newborn affected by other malpresentation, malposition and disproportion during labour and delivery	P03.1
**Foetus affected by maternal hypertensive disorders or diabetes**	Foetus and newborn affected by breech delivery and extraction	P03.0
	Preeclampsia/Eclampsia/ haemolysis, elevated liver enzymes, low platelet count (HELLP) syndrome	P00.0
	Pre-existing type 1 diabetes mellitus	O24.0
	Pre-existing type 2 diabetes mellitus	O24.1

Table S3. Frequency of stillbirths, neonatal and child deaths in the target population (HDSS) that are enrolled in the study and consented for MITS (4th February 2019 to 3rd February 2021).

Site	Age category	Events among HDSS residents	Mortality rate in HDSS (per 1000)	Deaths notified and eligible for the study	Deaths with consent for MITS
Ethiopia All Sites	Live births	11,017	-	-	-
	Stillbirths	173	15	164	116
	Neonatal deaths	132	12	145	59
	Infant/Child deaths	1,064	97	132	20
	TOTAL	1,369	124	441	195
Haramaya HDSS	Live births	3,210	3,210	-	-
	Stillbirths	32	10	45	41
	Neonatal deaths	6	2	22	11
	Infant/Child deaths	135	42	7	1
	TOTAL HARAMAYA	173	54	74	53
Harar HDSS	Live births	1,237	1,237	-	-
	Stillbirths	12	10	62	46
	Neonatal deaths	2	2	64	35
	Infant/Child deaths	23	19	12	3
	TOTAL HARAR	37	30	138	84
Kersa HDSS	Live births	6,570	6,570	-	-
	Stillbirths	129	19	57	30
	Neonatal deaths	124	19	59	12
	Infant/Child deaths	906	138	113	16
	TOTAL KERSA	1049	160	229	58

HDSS: Health & Demographic Surveillance System; CHAMPS: Child Health and Mortality Prevention Surveillance; MITS: Minimally Invasive Tissue Sampling

Figure S1: Death notification diagram

Figure S1: Death notification diagram

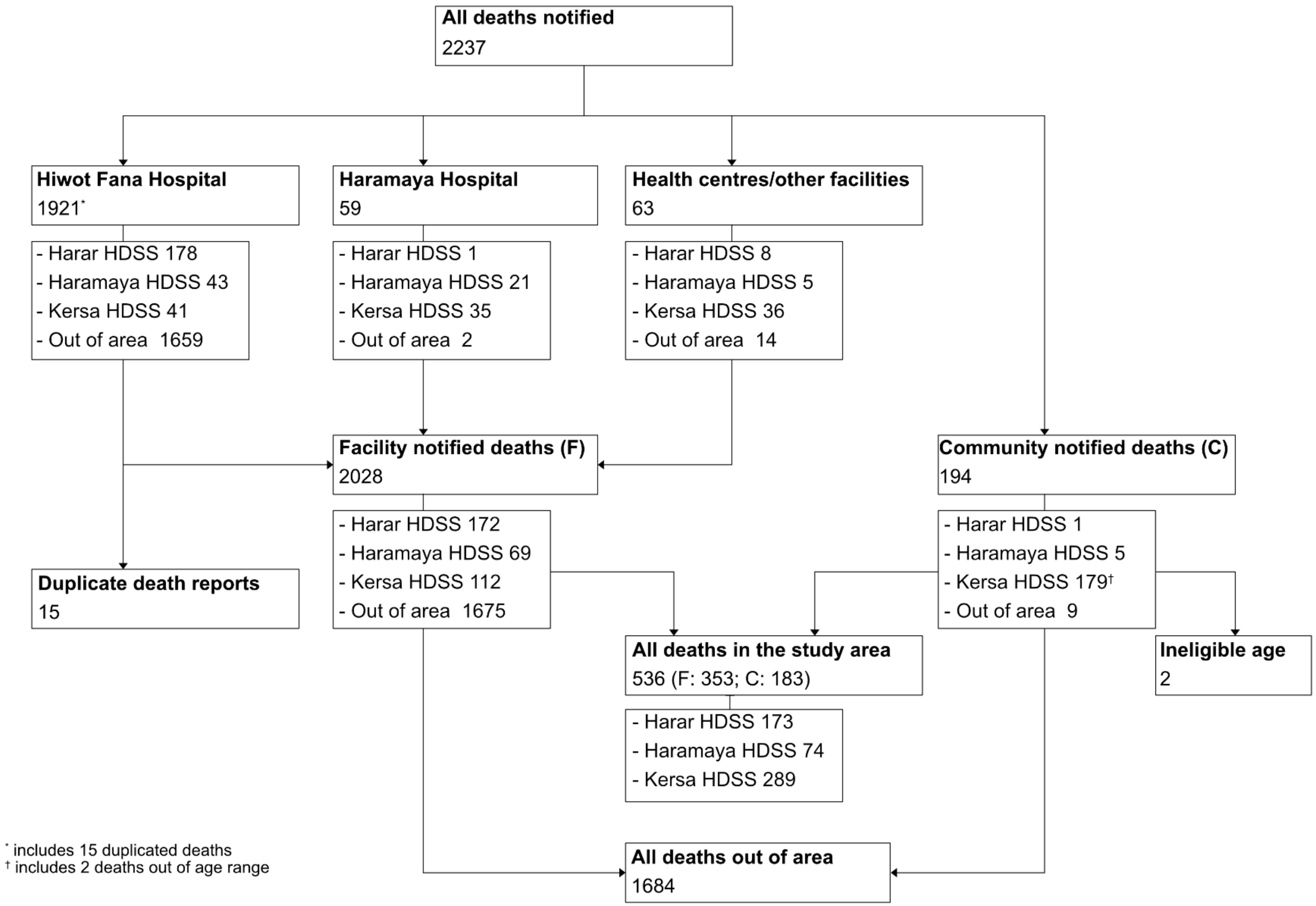

Table S4. Breakdown of consent by HDSS, age at death and location of death.

	**ALL HDSS (n=307)**			**Haramaya HDSS**			**Harar HDSS**			**Kersa HDSS**		
	**n**	**N**	**%**	**n**	**N**	**%**	**n**	**N**	**%**	**n**	**N**	**%**
**Age group**												
**Stillbirths**	116	139	**83**	41	44	**93**	46	61	**75**	30	34	**88**
**Neonates**	59	105	**56**	11	21	**52**	35	63	**56**	12	21	**57**
**Children 1-59 months**	20	63	**32**	1	3	**33**	3	9	**33**	16	51	**31**
**Location of death**												
**Facility**	175	264	**66**	52	66	**79**	83	132	**63**	39	65	**60**
**Community**	20	43	**47**	1	2	**50**	1	1	**100**	19	41	**46**
**All**	195	307	**64**	53	68	**78**	84	133	**63**	58	106	**55**

HDSS: Health & Demographic Surveillance System

Table S5. Maternal factors contributing the main cause of death found in 59 stillbirths

Main cause of stillbirth	Complications of placenta, cord, and membranes†		Maternal hypertensive disorders or diabetes		Malposition, malpresentation, disproportion, other complications of labour or delivery		Multiple pregnancy		Uterine rupture during delivery	
	n	%	n	%	n	%	n	%	n	%
Perinatal asphyxia or hypoxia	22	37	13	22	7	12	0	0	1	2
Neural tube defects	0	0	0	0	1	2	0	0	0	0·0
Other congenital birth defects	0	0	0	0	0	0·0	0	0	1	2
Small for gestational age	3	5	5	8	0	0·0	2	3	0	0
Sepsis	1	2	0	0	0	0·0	1	2	0	0
Undetermined	1	2	1	2	0	0·0	0	0	0	0
TOTAL (N=59)	27	46	19	32	8	13	3	5	2	4

†*Placenta available for 47 stillbirths among 114 (41%) included in this analysis.*
The denominator to calculate the % for each figure is N=59, the number of stillbirths were a maternal contributing factor was found.

Figure S2A: Categories of all identified causes of death combined for stillbirths, neonates, and children under 5 years

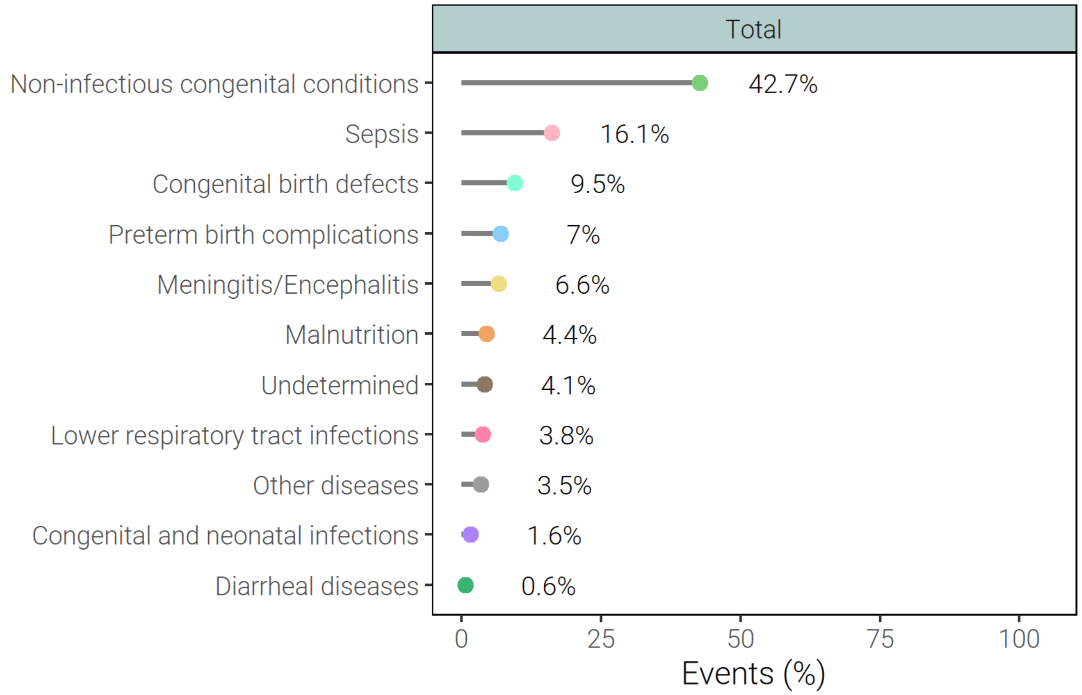

Non-infectious congenital and neonatal conditions: includes intrauterine hypoxia, birth asphyxia, low birth weight, meconium aspiration syndrome, light/small for gestational age, hydrops fetalis, apnoea and kernicterus.
Congenital birth defects: includes neural tube defects, congenital hydrocephalus, chromosomal abnormalities and other unspecified congenital malformations.
Preterm birth complications: includes prematurity, respiratory distress syndrome, necrotizing enterocolitis of foetus and newborn, intraventricular haemorrhage and neonatal jaundice of prematurity.
Others: includes diseases that are not very common, such as portal hypertension, hepatic failure, measles, hypovolemic shock, disseminated intravascular coagulation, febrile convulsions, hypothermia, anaemia, hypoglycaemia, hydrocephalus, cellulitis and accidental suffocation.

Figure S2B: Categories of all identified causes of death and position in the causal chain, combined for stillbirths, neonates, and children under 5 years

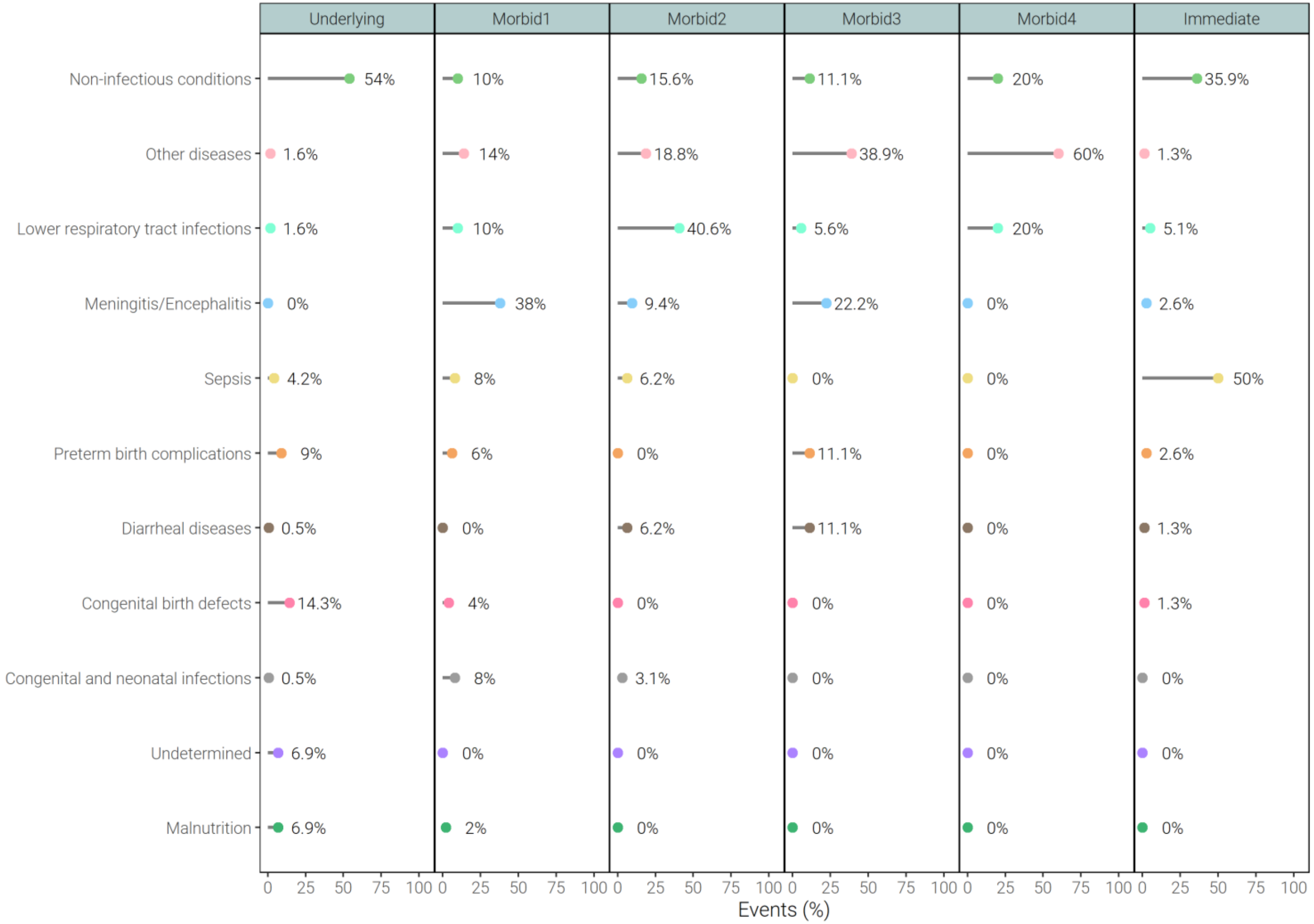

When more than one condition contributing to death is determined, the entire chain of events is documented; this included the underlying, comorbid conditions (in the causal pathway between underlying and immediate) and immediate factors. The DeCoDe procedure permits one underlying cause of death and one immediate cause but it allows multiple comorbid causes if identified.

Figure S3. Causal chain of events of all identified causes of death, combined for stillbirths, neonates, and children under 5 years

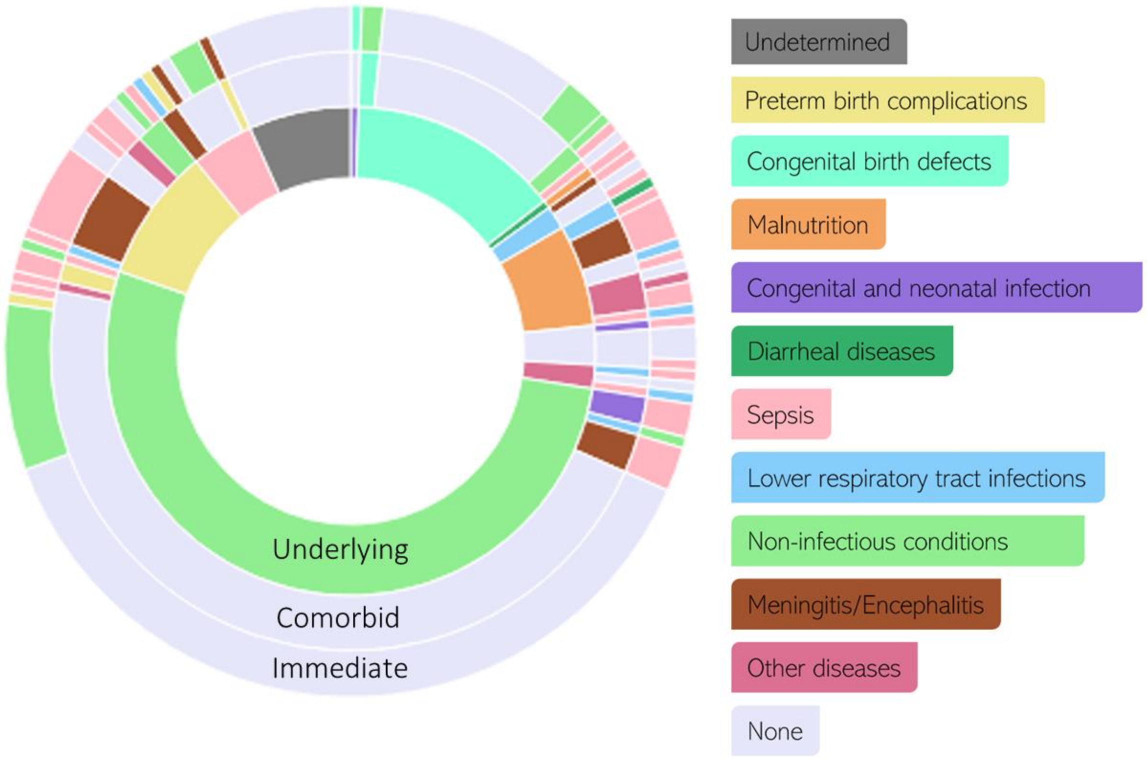

All cases must have an underlying cause. This underlying cause may be the unique cause of death identified or may trigger a chain of events. This included the underlying cause (one per case), comorbid conditions -in the causal pathway between underlying and immediate- (none, one or multiple for each case) and immediate cause of death (none or one per case).
